# Idiopathic Hypertrophic Cranial Pachymeningitis With Chiari Type I Malformation: Case Report and Review of the Literature

**DOI:** 10.7759/cureus.28466

**Published:** 2022-08-27

**Authors:** Aisha Khalid, Enoch O Uche

**Affiliations:** 1 Research, Harvard Medical School, Boston, USA; 2 Neurological Surgery, University of Nigeria Teaching Hospital, Enugu, NGA

**Keywords:** idiopathic, surgical decompression, refractory headache, chiari type i malformation, pachymeningitis

## Abstract

Idiopathic hypertrophic cranial pachymeningitis (IHCP) is a rare chronic inflammatory disease characterized by diffuse thickening of the dura mater. Although IHCP mostly presents as a diffuse lesion, it may also occur as focal tumour-like lesions. Here we present the first reported case of IHCP associated with a Chiari type I malformation (CMI). A 65-year-old man presented with a one-year history of chronic headache and vertigo exacerbated by standing and neck flexion. The neurological examination was unremarkable except for tongue wasting and fasciculations. MRI demonstrated features of CMI and findings suggestive of IHCP.

Posterior fossa decompression resulted in significant symptomatic improvement and the diagnosis of IHCP was confirmed on histopathology. Though there is no consensus about the management of IHCP in this case, we advocate surgical decompression with prolonged steroid therapy.

## Introduction

Idiopathic hypertrophic cranial pachymeningitis (IHCP) is a comparatively rare chronic inflammatory disorder associated with diffuse or localized thickening of the cranial dura mater and compression of adjacent structures with subsequent neurological deficits [1-2]; it predominantly affects males in their sixth and seventh decades of life [3]. Hypertrophic cranial pachymeningitis has been associated with many secondary diseases and syndromes such as infections, malignancy, and systemic autoimmune disease [4]; however, the exact etiology remains undetermined most of the time, hence the name IHCP [5]. The diagnostic findings in IHCP may include mild to moderate C-reactive protein (CRP) elevation with aseptic inflammatory cerebrospinal fluid (CSF) changes [6]. The sensitivity and specificity of CT and MRI in the diagnosis of IHCP are low, and there is currently no consensus regarding the definite treatment of patients [7]. Although steroids have been shown to offer significant symptom relief in most cases of IHCP, the improvement is transient, and issues of steroid dependence have been reported [8]. The combined therapy of steroids with immunomodulator therapies has also been reported to be effective in treating IHCP [1-8]. A case of IHCP occurring in association with Chiari type I malformation (CMI) is presented in our index report. To our knowledge, this is the first such report in the medical literature. Patient consent was obtained for the use of his demographic details and clinical findings for publication.

## Case presentation

**Figure 1:**
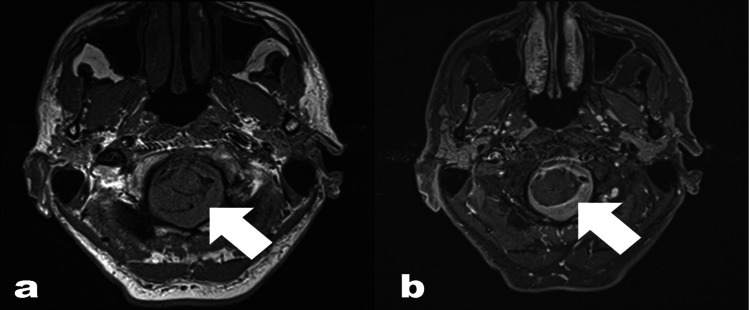
(A): Axial non-contrast T1 weighted spin echo MRI image of the brain showing asymmetric, smooth, abnormal pachymeningeal thickening at the foramen magnum (arrow), more pronounced on the left side. (B): Axial post-gadolinium contrast T1 weighted spin echo MRI image at the same location demonstrating avid enhancement of the pachymeningeal thickening (arrow).

**Figure 2:**
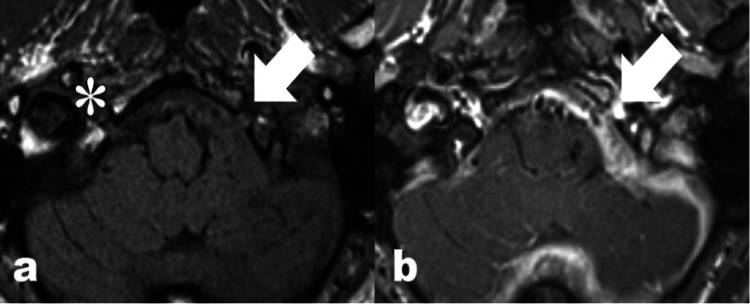
(A): Axial non-contrast small-field-of-view T1 weighted spin echo MRI image of the posterior fossa showing involvement of the left hypoglossal canal by abnormally thickened pachymeninges (arrow). The normally appearing right hypoglossal canal is marked with an asterisk for comparison. (B): Axial post-gadolinium contrast small-field-of-view T1 weighted spin echo MRI imaging at the same location demonstrating avid enhancement of the pachymeningeal thickening (arrow).

**Figure 3:**
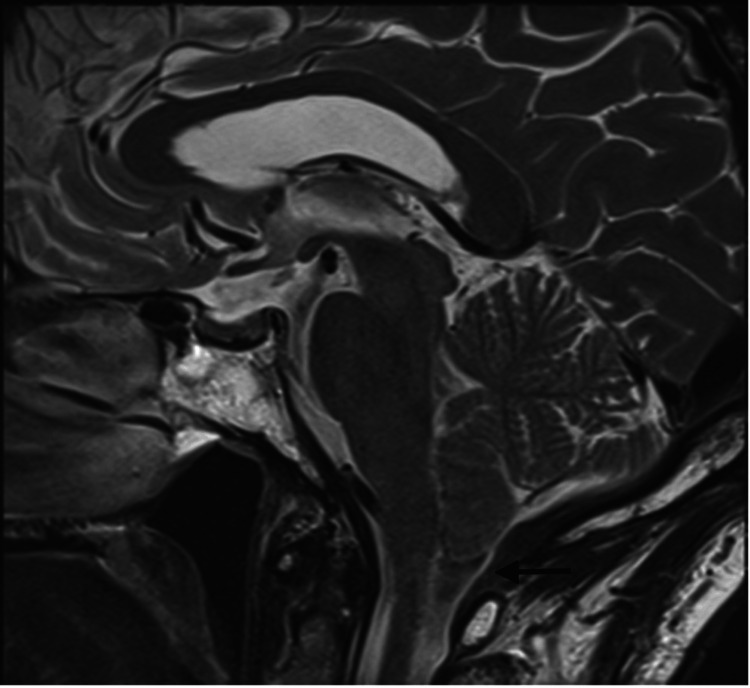
Sagittal T2 weighted spin echo MRI image of the midline brain demonstrating descent of the cerebellar tonsils 6 mm below the foramen magnum in keeping with Chiari type I malformation (arrow)

A 65-year-old Caucasian male presented with a one-year history of progressive headache and vertigo associated with upright posturing and neck flexion that worsened with coughing and sneezing. He had no travel history, weight loss, or family history of any genetic or immunological disorders. Neurological examination was unremarkable except for fasciculations and wasting of the left side of his tongue. Inflammatory markers were very mildly elevated, and an autoimmune screen, syphilis and malignancy screen were negative. A lumbar puncture was done and CSF examination was inconclusive. A CT scan revealed a low-lying right cerebellar tonsil suggestive of a CMI without any evidence of hydrocephalus. An MRI examination of his whole neuraxis was subsequently performed, demonstrating prominent pachymeningeal thickening in the region of the foramen magnum as well as cerebellar tonsillar descent 6 mm below the foramen magnum, consistent with IHCP and an associated CMI (Figures 1-3).

A posterior fossa decompression was performed including excision of the posterior arch of C1 and augmentation duraplasty. The dura beneath the occiput at the foramen magnum was extremely thick and tough, but otherwise unremarkable in appearance, and specimens were sent for histopathological examination post operatively. Microscopically, in the post-operative sample, there was a non-specific, variable chronic inflammatory cell infiltrate, lymphoplasmacytic cell infiltrates, and hyaline degeneration. Duraplasty was completed with a commercially available dural substitute, and the patient made an unremarkable post-operative recovery with good resolution of his pre-operative symptomatology.

A follow-up for 12 months showed complete recovery and relief of his symptoms.

## Discussion

IHCP is a rare disorder of unknown etiology. The spinal form of idiopathic hypertrophic pachymeningitis was first described in 1869 by Charcot and Jaffrey while Nafziger and Stern described the first cranial case in 1949 [9-10]. It is associated with inflammatory fibrosis that causes diffuse thickening of the dura mater [11]. Various disease associations have been recognized, including metabolic disorders, sarcoidosis, infections (tuberculosis, human T-lymphotropic virus, syphilis, meningococcal meningitis), immunosuppression [12-13] as well as certain autoimmune and rheumatological disorders (Wegener granulomatosis, rheumatoid arthritis, multifocal fibrosclerosis, and mixed connective tissue disorder) [14]. Other rarer associations with IHCP include synovitis, acne, pustulosis, hyperostosis, osteitis (SAPHO) syndrome [15], Tolosa-Hunt syndrome [16], and pseudotumor cerebri [17]. The differentials of IHCP include meningiomas, tuberculous meningitis, neurosarcoidosis, and lymphomas [17]. This index case is the first report of an association with a CMI.

The most common anatomical location of thickened dura-mater is the posterior fossa in the cranium, in IHCP, hence it could cause non-communicating hydrocephalus [18].

Headache is the most reported symptom and, in some cases, may remain the only presenting symptom for years [19]. In a retrospective cohort of patients, it was reported in 82%-98% of people with IHCP [20] and is thought to be likely attributable to dura mater inflammation [21]. The second most common symptom experienced by patients with IHCP is cranial nerve palsies in 60%-81% [22] of which the oculomotor nerve is the most reported [23], Involvement of the optic nerve in IHCP is quite rare but can lead to unilateral or bilateral painless loss of vision [24]. Other involved cranial nerves with equal frequency are IV, V, VIII, IX, X, and XII [25]. These CN findings result from ischemia resulting in neural compression, epineural inflammatory infiltrates and thickened meningeal encroachment [26]. Other common symptoms are cerebellar ataxia and seizures [27].

IHCP is a diagnosis of exclusion [28]. However, a recent approach that includes advanced neuroimaging and histopathological examination is useful for making a diagnosis of IHCP while excluding other secondary causes [29]. Contrast-enhanced MRI is superior to contrast-enhanced CT, in the diagnosis of IHCP [30], and showed its usefulness in the diagnosis, monitoring of disease progression, and treatment follow-up [31]. On MRI, hypertrophic dura mater appears hypointense on T1-weighted image (T1WI) and hypointense on T2-weighted image (T2WI) with or without a hyperintense edge [32]. The gold standard for the diagnosis of IHCP is the histopathological evaluation of a dural biopsy specimen which shows chronic inflammatory changes with fibrous tissue hyperplasia and concentric arrangement in some cases as well as cellular infiltrates of lymphocytes, fibroblasts, and plasma cells [33]. Microscopic examination in some cases shows non-specific exuberant fibroplasia and focal hyaline degeneration [34]. IHCP can be misdiagnosed as a primary headache of unknown etiology when the dura mater is not thickened enough on a plain MRI or CT scan or acute subdural hematoma [34-5]. In our considered opinion, whether IHCP represents chronic inflammatory sequelae of the pathogenetic events occurring in the posterior fossa in association with CMI or an independent lesion remains to be elucidated. 

The treatment of IHCP remains controversial. Despite a lack of consensus about the duration and the dose of steroid therapy, they are effective in alleviating the symptoms of IHCP [34]. The combined therapy of immunomodulators and steroids has been found to be effective [35]. But still, no clinical trial has been done due to low evidence of the disease to date. Surgical decompression as a treatment option is reserved for patients with evidence of mass effects due to thickening of skull base dura refractory to all medical management or obstructive hydrocephalus [36].

## Conclusions

We have reported a case of IHCP occurring in association with CMI. We advocate a heightened clinical suspicion for IHCP in patients with Chiari malformation. Diagnostic neuroimaging and laboratory evaluation should be performed for patients with suggestive neurological presentations. Ultimately, the definitive diagnosis of IHCP is confirmed by salient findings on histopathological evaluation of dural biopsy specimens. Posterior fossa decompression and treatment with steroids were rewarded with success in the management of our index patient and are hereby recommended. Long-term observation will be required to monitor the overall outcome of our index case.
